# Increasing incidence of *Dirofilaria immitis* in dogs in USA with focus on the southeast region 2013–2016

**DOI:** 10.1186/s13071-018-2631-0

**Published:** 2018-01-17

**Authors:** Jason Drake, Scott Wiseman

**Affiliations:** 1 Elanco Animal Health, 2500 Innovation Way, Greenfield, IN 46140 USA; 2 Elanco Animal Health, Basingstoke, Hants UK

**Keywords:** *Dirofilaria immitis*, Incidence, United States of America, Dog, Heartworm

## Abstract

**Background:**

A recent American Heartworm Society (AHS) survey on the incidence of adult heartworm infections in dogs in the United States of America showed a 21.7% increase in the average cases per veterinary clinic from 2013 to 2016. The analysis reported here was performed to see if heartworm testing results available via the Companion Animal Parasite Council (CAPC) aligned with the AHS survey and whether changes in heartworm preventive dispensing accounts for the increased incidence. The resistance of *Dirofilaria immitis* to macrocyclic lactones (MLs) has been previously reported.

**Methods:**

An analysis of 7–9 million heartworm antigen tests reported annually to the Companion Animal Parasite Council (CAPC) from 2013 to 2016 was conducted and compared to the 2016 AHS survey. A state-by-state analysis across the southeastern USA was also performed. National heartworm preventive dispensing data were obtained from Vetstreet LLC and analyzed. All oral, topical and injectable heartworm preventives were included in this analysis, with injectable moxidectin counting as six doses.

**Results:**

Positive antigen tests increased by 15.28% from 2013 to 2016, similar to the 21.7% increase reported by the AHS survey. Incidence in the southeastern USA increased by17.9% while the rest of USA incidence increased by 11.4%. State-by-state analysis across the southeastern USA revealed an increased positive test frequency greater than 10% in 9 of 12 states evaluated. During this time, the overall proportion of dogs receiving heartworm prophylaxis remained relatively unchanged. Approximately 2/3 of the dogs in the USA received no heartworm prevention each year.

**Conclusion:**

These CAPC data show the rate of positive heartworm tests increasing significantly (*P* <  0.0001) in the USA from 2013 to 2016, with a higher rate of increase in the southeastern USA than nationally. Only 1/3 of dogs in the USA were dispensed one or more doses of heartworm prevention annually by veterinarians, averaging 8.6 monthly doses/year. Veterinarians and pet owners should work together to follow CAPC and AHS guidelines to protect dogs from infection with *D. immitis*. Lack of preventive use and the emergence of heartworm resistance to MLs could both be impacting the increased rate of positive heartworm tests in dogs.

## Background

Canine heartworm disease caused by *Dirofilaria immitis* can be found throughout the United States of America [1]. Prevention of *D. immitis* via the daily administration of diethylcarbamazine (DEC) shifted to monthly administration of macrocyclic lactones (ML) containing drug products following FDA approval of ivermectin (Heartgard-30®, Boehringer Ingelheim, Duluth, GA, USA) in 1987 and milbemycin oxime (Interceptor®, Elanco, Greenfield, IN, USA) in 1990. Additional preventive products, all containing ML, have entered the market over the past three decades. Organizations like AHS and CAPC have been working to raise public awareness of HW disease and have provided recommendations for regular adult HW antigen testing and year-round heartworm prevention for many years [2, 3]. The veterinary profession in the USA has embraced the importance of preventing HW disease, encouraging pet owners to follow the recommendations of organizations like AHS and CAPC. While millions of dogs across the USA are tested for the presence of adult *Dirofilaria immitis* and are prescribed MLs as preventives, many of the over 70 million dogs in the USA [4] go unprotected. In 2013, approximately 7 million canine heartworm antigen tests were performed by commercial diagnostic laboratories that reported data to CAPC and made available online via CAPC maps [5]. CAPC estimates that 30% of the testing results in the USA are reported to CAPC via these laboratories [6]. If this estimate is correct, a total of 23 million HW antigen tests would have been performed in 2013, leaving almost 50 of the 70 million dogs in the USA untested for heartworms.

An analysis of lack of effectiveness (LOE) reports to the FDA conducted in 2005 showed an increase in the number of HW product efficacy complaints during 2000–2003 [7]. Elanco Animal Health-funded research during 2007–2010 attempting to identify the underlying cause of this increase, ultimately uncovering evidence of emerging resistance of *D. immitis* to MLs through clinical laboratory and field study failures of heartworm preventives and identification of genetic markers for resistance [8].

AHS has historically conducted a triennial HW survey in the USA, publishing HW incidence maps every three years since 2001 [9]. Following the 2016 survey, AHS reported a 21.7% increase in the average number of dogs diagnosed positive for adult HW per clinic in 2016 [10].

To further investigate the changing incidence of canine HW infection in the USA, this study analyzed the reports of positive *D. immitis* antigen test results in the USA during 2013–2016 to determine if the antigen test results performed upon millions of blood samples from dogs corroborated the survey reports gathered by AHS.

## Methods

Data including the total number of heartworm tests performed and the total number of antigen-positive test results were collected from the CAPC maps available online [11]. CAPC state [6] that data in the maps were provided by IDEXX Laboratories and ANTECH Diagnostics, that these data were statistically significant, and serve as a strong representation of the parasite activity for each area. However, CAPC also caution that these data do not represent the total number of positive tests. Instead, CAPC estimates they represent less than 30% of the activity in the geographical regions. Based on the large annual sample size, we consider these data representative of the overall national situation regarding HW testing and infection rates in dogs.

Total canine HW test results were obtained for the USA as well as for the following 12 individual states from the southeast region: Texas, Oklahoma and Arkansas, where early investigations regarding resistance to MLs were conducted [12]; Louisiana, where evidence of resistance to ivermectin was documented with 2 separate isolates [13]; Missouri, Tennessee, Mississippi, Alabama, North Carolina, South Carolina, Georgia and Florida. The southeastern USA was given additional focus in this analysis as it was the area initially identified as having the highest number of heartworm product complaints about LOE reports by the FDA in 2005 [7]. Using 2013 as a baseline, the increase in incidence was calculated for each year from 2014 to 2016. The additional context was provided by examining the percent annual increase in the number of tests reported and the percent annual increase in the number of positive cases. This enabled an assessment of whether the increase in positive results was occurring in line with the increase in the number of tests reported or whether the positive results were increasing at a faster rate. To assess whether the incidence was consistently changing over time regression lines were also fitted.

The statistical significance of changes in incidence compared to 2013 were assessed through *χ*^2^ tests by constructing 2 × 2 contingency tables of positive and negative results for each year. Based on available data, this method was considered the most appropriate approach; however, two potential weaknesses need to be highlighted. First, the method assumes mutual statistical independence of all data points. This is likely, not satisfied since, based on testing guidelines, dogs will be represented more than once within the dataset. However, it is not possible to calculate the degree of correlation between observations since data are only available at the yearly summary level and not at the individual dog level. Secondly, the extremely large sample size means that the test will be able to detect very small differences. Potentially, differences so small as to be normally considered inconsequential could be detected and declared significant. Therefore the clinical relevance and significance need to be assessed to provide full context.

To assess the impact of prescribing habits on the incidence rate, analysis of proprietary dispensing data from Vetstreet LLC, Trevose, Pennsylvania (National Estimate, Practice Dispensing Data, 2013–2016, E-168) from 2013 to 2016 were obtained from Vetstreet and the trends examined. These data are obtained by Vetstreet through access to the practice management software utilized by approximately 7000 veterinary hospitals across the USA. Vetstreet is then able to estimate the total market for companion animal hospitals. These data include the estimated number of dogs treated with a heartworm preventive, the estimated number of doses of heartworm preventive doses dispensed, and the average number of doses per year dogs are receiving. All oral, topical and injectable heartworm preventives were included in the analysis, with injectable moxidectin counting as 6 doses.

## Results

Across the USA, close to 7 million canine antigen tests were performed in 2013 by the commercial diagnostic laboratories that reported data to CAPC. The total number of test results captured increased each year to a total of more than 9.2 million tests in 2016. The overall incidence of positive *D. immitis* antigen tests in the USA increased each year, with a national incidence of 1.11% (2013), 1.18% (2014), 1.23% (2015) and 1.28% (2016). The increase in incidence compared to 2013 was statistically significant for each year (*P* <  0.0001; Tables 1, 2 and 3).

**Table 1:** Summary of incidence of positive heartworm antigen test results and the relative change in incidence 2013 to 2016

State/Region	2013			2016			Relative change in incidence 2016 vs 2013 (%)
	No. of tests conducted	No. of positive results	Incidence (%)	No. of tests conducted	No. of positive results	Incidence (%)	
United States	6,980,504	77,557	1.11	9,266,427	118,689	1.28	15.28
Alabama	84,958	2481	2.92	114,531	4428	3.87	32.39
Arkansas	36,283	1259	3.47	55,482	2405	4.33	24.92
Florida	594,004	7979	1.34	712,653	9048	1.27	-5.48
Georgia	241,678	5348	2.21	317,229	7842	2.47	11.71
Louisiana	61,321	3782	6.17	103,667	7242	6.99	13.27
Mississippi	16,017	1340	8.37	38,579	2905	7.53	-9.99
Missouri	145,571	1437	0.99	168,206	2549	1.52	53.51
North Carolina	324,493	6555	2.02	441,015	9386	2.13	5.36
Oklahoma	44,351	831	1.87	60,327	1363	2.26	20.58
South Carolina	136,549	3099	2.27	176,438	4550	2.58	13.63
Tennessee	140,553	3423	2.44	173,571	5326	3.07	26.00
Texas	525,667	14,322	2.72	735,395	23,489	3.19	17.23
Southeastern USA	2,351,445	51,856	2.21	3,097,093	80,533	2.60	17.91
Rest of USA	4,629,059	25,701	0.56	6,169,334	38,156	0.62	11.40

**Table 2:** Relative change in incidence of positive heartworm antigen test results in 2014–2016 compared to 2013 and test statistics for the change in incidence

State/Region	Relative change in incidence % [*χ*^*2*^ (*P*-value)]		
	2014 vs 2013	2015 vs 2013	2016 vs 2013
United States	5.83% [136.86 (< 0.0001)]	10.68% [470.88 (< 0.0001)]	15.28% [961.93 (< 0.0001)]
Alabama	11.78% [17.65 (< 0.0001)]	16.89% [37.22 (< 0.0001)]	32.39% [130.54 (< 0.0001)]
Arkansas	12.59% [10.37 (0.0013)]	29.16% [55.71 (< 0.0001)]	24.92% [42.80 (< 0.0001)]
Florida	-2.57% [2.79 (0.0946)]	-1.26% [0.70 (0.4027)]	-5.28% [13.66 (0.0002)]
Georgia	11.08% [34.38 (< 0.0001)]	14.28% [58.39 (< 0.0001)]	11.71% [39.99 (< 0.0001)]
Louisiana	3.28% [2.45 (0.1176)]	10.52% [25.79 (< 0.0001)]	13.27% [41.38 (< 0.0001)]
Mississippi	-8.37% [5.77 (0.0163)]	-3.19% [0.90 (0.3425)]	-9.99% [11.03 (0.0009)]
Missouri	18.73% [23.53 (< 0.0001)]	35.19% [80.16 (< 0.0001)]	53.51% [173.63 (< 0.0001)]
North Carolina	0.58% [0.12 (0.7287)]	4.30% [6.84 (0.0089)]	5.36% [10.73 (0.0011)]
Oklahoma	6.43% [1.80 (0.1796)]	4.27% [0.85 (0.3553)]	20.58% [18.53 (< 0.0001)]
South Carolina	11.19% [19.50 (< 0.0001)]	9.97% [16.47 (< 0.0001)]	13.63% [30.89 (< 0.0001)]
Tennessee	10.46% [18.99 (< 0.0001)]	28.05% [130.90 (< 0.0001)]	26.00% [114.97 (< 0.0001)]
Texas	2.77% [6.12 (0.0134)]	9.03% [65.22 (< 0.0001)]	17.23% [232.36 (< 0.0001)]

**Table 3:** Statistics for fitted regression lines to assess the trend in incidence of positive heartworm antigen test results across 2013–2016

State/Region	*R* ^2^	*F-*value	*P*-value
United States	0.9968	631.16	0.0016
Alabama	0.9667	58.03	0.0168
Arkansas	0.8043	8.22	0.1032
Florida	0.6924	4.50	0.1679
Georgia	0.6111	3.14	0.2183
Louisiana	0.9681	60.63	0.0161
Mississippi	0.4810	1.85	0.3065
Missouri	0.9994	3505.81	0.0003
North Carolina	0.9186	22.56	0.0416
Oklahoma	0.7444	5.82	0.1372
South Carolina	0.7296	5.40	0.1458
Tennessee	0.8590	12.18	0.0732
Texas	0.9573	44.89	0.0216

**Fig. 1:**
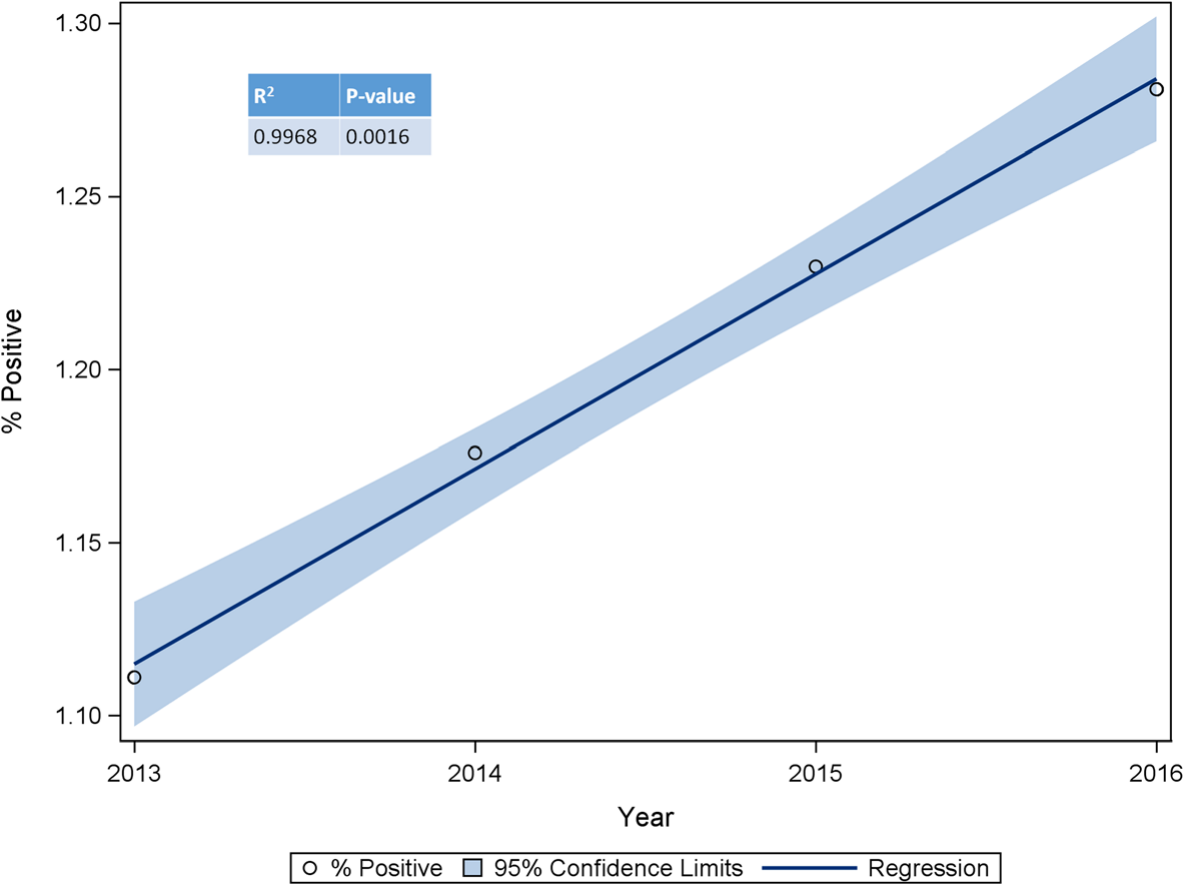
Percentage of positive heartworm antigen test results by year with fitted regression line and 95% confidence limits

The trend for increasing incidence of positive results can be seen in Fig. 1. A regression line was fitted to examine this trend (*R*^2^ = 0.9968; *F*_(1,2)_ = 631.16, *P* = 0.0016) and revealed that the incidence of positive HW antigen results increased by approximately 0.056% year-on-year. If this increase continues at a constant rate, more than 1.5% of tests would be positive in 2020. If the number of tests conducted per year were to remain at the 2016 level, this would represent approximately 140,000 positive results in 2020. However, when taking into account the increase in the number of tests conducted each year, the figure would likely be higher.

This trend represents a relative percent increase in *D. immitis* incidence compared to 2013 of 5.84% in 2014, 10.68% (2015) and 15.28% (2016), corroborating the 21.7% increase reported nationally between 2013 and 2016 by AHS [10]. The results of 9.2 million tests were reported in 2016 representing a 33% increase compared to the 7.0 million test results reported in 2013. In the same period, the number of positive test reports increased by 53% from 77,557 (2013) to 118,689 (2016). The positive reports are increasing at a notably faster rate than the increase in test result reporting.

Vetstreet data estimated the total number of dogs visiting veterinarians annually as just under 53 million in 2013, rising to just over 56 million in 2016. Although the number of dogs receiving HW preventive increased by 650,000 from 19.3 million in 2013 to 20.0 million in 2016, the proportion of dogs receiving HW preventive decreased year on year from 36.68% in 2013 to 35.69% in 2016. When a regression line was fitted to these data, a significantly decreasing trend (*R*^2^ = 0.9723; *F*_(1,2)_ = 70.13, *P* = 0.0140) was noted. This provides a worrying indication that the proportion of dogs receiving HW preventives is decreasing year-on-year nationally. The average number of doses given to dogs remained static (approximately 8.6 doses/year). However, the missed opportunity for prevention is significant. If the 2013 level of 36.68% of dogs receiving HW prevention had been maintained, instead of dropping to 35.69% in 2016, more than half million additional dogs would have received preventive treatments.

**Fig. 2:**
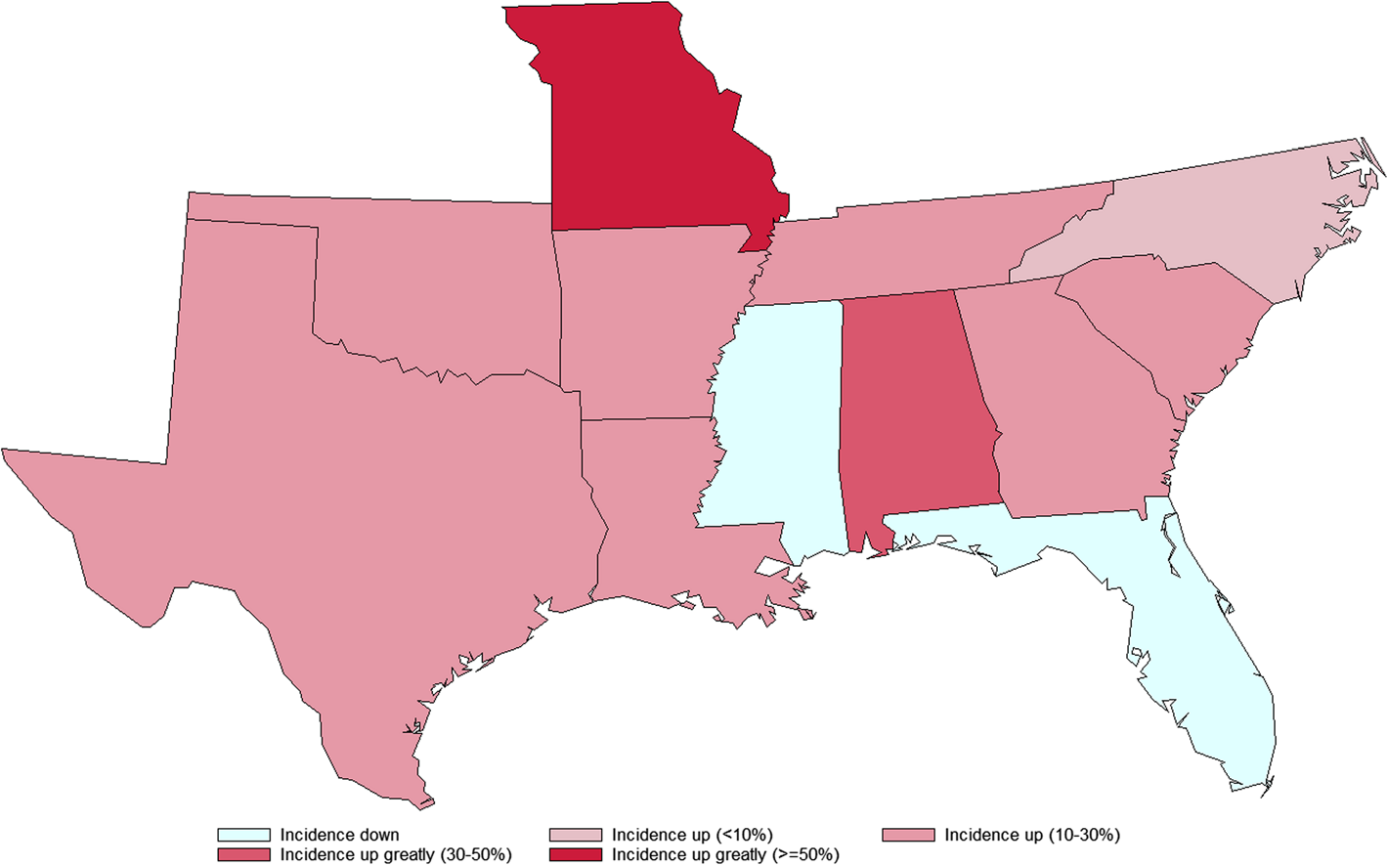
Comparison of incidence of positive heartworm antigen test results in the southern United States 2013 vs 2016

When 12 individual states in the southeastern USA are considered, the results reflect the national trend. Incidence in the southeastern USA increased 17.9% while the rest of the USA incidence increased by 11.4%. The incidence rate in 2016 was higher compared to 2013 in 10 out of the 12 states (Table 1). In each case, the increase was statistically significant (*P* <  0.0001, Table 2). The relative change in incidence was categorized (decrease, increase by < 10%, increase by 10–30%, increase by 30–50% and increase > 50%) and shown in Fig. 2. In spite of the total number of positive antigen test results increasing between 2013 and 2016 in Florida and Mississippi the incidence in both states went down. In the state with the highest overall HW incidence, Mississippi, the incidence of positive results reduced from 8.37% in 2013 to 7.53% in 2016. In Florida, the incidence decreased from 1.34% in 2013 to 1.27% in 2016, becoming the only state in the southeast below the national HW incidence level of 1.28%. The results from Texas provide an interesting contrast to Florida. The two states had a similar number of tests conducted throughout the period of interest. Whilst the incidence of positive results in Florida remained relatively static year on year (1.34%, 1.31%, 1.33% and 1.27% in 2013, 2014, 2015 and 2016, respectively), in Texas the incidence increased year on year (2.72%, 2.80%, 2.97% and 3.19% in 2013, 2014, 2015 and 2016, respectively).

When the trend across time in the individual states was examined through statistical regression, significant (*P* <  0.05) increasing trends were observed in 5 of the 12 states (Alabama, Louisiana, Missouri, North Carolina and Texas; see Table 2). In Missouri in particular, the correlation between incidence and year was very strong (*R*^2^ = 0.9994). The number of positive cases reported in Missouri remained relatively low compared to the other states studied (1437, 1745, 2173 and 2549 in 2013, 2014, 2015 and 2016, respectively) but the relative change from 2013 to 2016 was the highest seen in any of the states studied (53.5%).

## Discussion

Data, as presented in the CAPC maps, showed a 2016 national increase in antigen-positive test status vs 2013 of 15.28% which is similar to the HW survey results reported by the AHS of a 21.7% increase in average cases per clinic between 2013 and 2016 [10]. Incidence in the southeastern USA increased 17.9% while the rest of the USA incidence increased by 11.4%. Based on both this analysis and the AHS survey findings, the incidence of HW across the USA appears to be slowly increasing, despite the widespread commercial availability of prescription HW preventives. Incidence in the southeastern states appears to be increasing at a higher rate than the rest of the USA. There could be many reasons for this, including decreased usage of preventives, poor compliance with heartworm prevention guidelines, etc. However, the impact of emerging resistance to ML should not be discounted. The rate of increase in the incidence of HW positive status is greater in Arkansas and Louisiana (where evidence of resistance was first documented) than the average increase nationally, while the incidence has dropped in Florida, over that same 2013–2016 time frame, where evidence of resistance has yet to be confirmed. The emergence of heartworm resistance to ML prompted CAPC to revise their heartworm prevention guidelines in 2013, recommending “immediate and aggressive” treatment of infected dogs with melarsomine [14]. Avoidance of the “slow-kill” practice of giving ML to heartworm positive dogs is recommended since this can increase the percentage of circulating microfilaria with genetic markers of resistance [8, 14].

At first glance, the data from the southeastern USA suggest an issue of regional importance. However, movement of dogs across the USA from the southeast to other parts of the country is a common occurrence. During 2017, hundreds of animals were shipped from Louisiana, Texas and Florida to various parts of the USA including Pennsylvania, Maryland, Michigan and California following Hurricane Harvey and Hurricane Irma [15–19]. While these reported movements occurred after the increase in incidence described in this manuscript, it is possible that some of these dogs were infected with heartworm and may become sources of infection in areas with lower incidence than where they originated. Movement of dogs following hurricanes in the southeast has also occurred at earlier times including after Hurricane Katrina in 2005. A study of 414 dogs rescued from Hurricane Katrina and shipped from Louisiana to various states in the USA 48.8% were positive for heartworm [20].

Importation of dogs from highly endemic areas may spread both sensitive, and potentially ML resistant, heartworms into new areas at a faster rate than local biological spread via mosquitos alone.

## Conclusion

The incidence of heartworm infections in dogs appears to be increasing in the USA, with the incidence rates within the southeastern region including the states of Texas, Arkansas, Louisiana, Alabama and Georgia significantly exceeding the national rate and cases in Missouri increasing more than 50%. Mississippi maintained the highest percent incidence of any southeastern state, and the total number of positive test results in Mississippi more than doubled from 2013 to 2016. Veterinarians should continue to emphasize the importance of annual testing and year-round use of ML containing HW preventives as well as continue to follow AHS and CAPC guidelines regarding overall HW prevention strategies. An increased emphasis on the importance of HW prevention should be communicated to pet owners by all members of the veterinary healthcare team.

## Abbreviations

AHS: American Heartworm Society
CAPC: Companion Animal Parasite Council
FDA: US Food and Drug Administration
HW: Heartworm
LOE: Lack of effectiveness
MLs: Macrocyclic lactones
